# Circulating Nitrite and Nitrate are Associated with Job-Related Fatigue in Women, but not in Men 

**DOI:** 10.3390/ijerph10072813

**Published:** 2013-07-05

**Authors:** Jiro Takaki

**Affiliations:** Department of Public Health and Occupational Medicine, Mie University Graduate School of Medicine, 2-174 Edobashi, Tsu, Mie 514-8507, Japan; E-Mail: jirosinryounaika-tky@umin.ac.jp; Tel.: +81-59-232-1111 (ext. 6372); Fax: +81-59-231-5012

**Keywords:** interaction, demands-control model, fatigue, gender difference, job strain, nitric oxide

## Abstract

A recent study indicated that serum nitrite and nitrate (NO_x_) is inversely associated with general fatigue. The purpose of this study was to confirm the negative association between nitric oxide (NO) and fatigue and to examine whether NO can prevent fatigue caused by job strain. The subjects, 570 workers (272 men and 298 women), answered self-administered questionnaires and underwent a medical examination. Job strain was measured using the Job Content Questionnaire. Fatigue was evaluated using the Profile of Mood States. Venous blood samples were collected after overnight fasting. Plasma NO_x_ concentration was determined by the ozone-based chemiluminescence assay. Plasma NO_x_ levels were significantly (*p* < 0.05) negatively associated with fatigue even after adjustment for job strain and potential confounders in women, but not in men. Significant (*p* < 0.05) interactions showed that, in women, as the level of the job strain worsened, fatigue was exacerbated, but the plasma NO_x_ seemed to buffer the association, even after adjustment for potential confounders and the interaction between job strain and vegetable intake. In women, NO seemed to be inversely associated with fatigue and to buffer the association between job strain and fatigue, but not in men.

## 1. Introduction

Nitric oxide (NO), the mediator of endothelium-dependent relaxation, is produced by the oxidation of L-arginine in an enzymatic reaction catalyzed by nitric oxide synthase (NOS). The measurement of NO itself is extremely difficult, because of its radical nature and very short half-life *in vivo*, most likely shorter than 0.1 s [1,2]. NO is instantly converted to nitrite (NO_2_^−^) and nitrate (NO_3_^−^) [1,2]. NOS activity can be determined only in tissue or cell homogenates [1]. Therefore, determination of stable end products of NO radical, NO_2_^−^ and NO_3_^−^ (NO_x_), in plasma is most often used as an index of systemic NO formation [1,2,3,4,5]. Plasma NO_x_ levels reflect not only NO production and NOS activity, but also an imbalance in oxidant/antioxidant mechanisms with reduced antioxidant defenses and the amount of ingested nitrates [3,5]. Excess visceral adiposity induces excess generation of reactive oxygen species (ROS) and diminished antioxidant defense mechanisms [6]. Cigarette smoke contains abundant ROS [7]. Excessive chronic exposure to alcohol usually results in decreased levels of antioxidant defenses [8]. It is well-established that exercise increases ROS formation in skeletal muscle [9]. To control for the imbalance in oxidant/antioxidant mechanisms and the amount of ingested nitrates, measurement should be made after overnight fast and obesity, cigarette smoking, alcohol consumption, and exercise should be adjusted for in the analysis. 

Recently, it has been indicated that serum NO_x_ concentrations are inversely associated with scores of general fatigue evaluated by the Multidimensional Fatigue Inventory, a self-rating scale [10], in healthy elderly subjects [3]. NO plays an essential role in vascular homeostasis, metabolic regulation, and immune processes [2,3]. NO can be cytoprotective under physiological and pathophysiological conditions [11,12]. In the central nervous system, NO production is associated with cognitive function, its role spanning from the induction and maintenance of synaptic plasticity to the control of sleep, appetite, body temperature and neurosecretion, and physiological amounts of NO are believed to be neuroprotective [13]. Moreover, NO has been hypothesized to have a role in the etiology of chronic fatigue syndrome [14]. Thus, physiological amounts of NO seem to play a role in prevention of fatigue in healthy individuals.

In this study, preventive effect of NO on fatigue in healthy workers was hypothesized. The inverse association between NO and fatigue was confirmed in working population using plasma NO_x_ levels. Moreover, in previous studies, high job strain evaluated using the demands–control model [15] seemed to cause fatigue [16,17,18]. Thus, whether NO can prevent fatigue caused by job strain was also assessed. Previous studies have suggested that there are gender differences in NO production/release or in responses to NO [19,20,21]. Thus, the data in men and women were analyzed separately.

## 2. Methods

### 2.1. Subjects

The subjects in this study were recruited from all the full-time workers (n = 1,003) at 16 organizations comprising a manufacturing company, an office of a distribution company, supermarkets, and a health care institution in Japan. The purpose and procedure of the survey were explained to the participants in the documents. Written informed consent was obtained from all participants. Approximately 2 weeks after distribution of the self-administered questionnaires, they were collected from 605 workers (response rate: 60.3%) at their medical examination. Because of missing data, 570 workers (272 men and 298 women; 19 workers in the manufacturing company, 138 workers in the office of a distribution company, 286 workers in the supermarkets, and 127 workers in the health care institution) were included in the analyses. This study was approved by the ethics committee of the Okayama University Graduate School of Medicine, Dentistry, and Pharmaceutical Sciences, and was performed according to the Declaration of Helsinki.

### 2.2. Parameters

#### 2.2.1. Job Strain

Job strain was measured using the Job Content Questionnaire (JCQ), developed by Karasek based on the demands-control model [15]. The JCQ includes scales for job demands (five items; range, 12–48) and job control (nine items; range, 24–96), with a four-point response option from 1 (strongly disagree) to 4 (strongly agree). The JCQ was translated into Japanese and the internal consistency reliability and factor- and construct-validity have been reported to be acceptable [22]. A job strain index, which is calculated as job demands divided by job control, has been used as an indicator of job strain; higher scores indicate greater strain [23]. To place inverse imbalance of the same magnitude (for example 0.5× and 2×) in the same distance from X (when job demands and job control are equivalent) the index was logarithmically transformed. 

#### 2.2.2. Fatigue

Fatigue was evaluated using the Profile of Mood States (POMS), which is a valid and reliable self-administered questionnaire that assesses the mood of a subject [24]. The POMS includes scales for fatigue (seven items; range, 0–28), with a five-point response option. The POMS was translated into Japanese and the validity and reliability have been confirmed to be excellent [25]. 

#### 2.2.3. Plasma NO_x_

Venous blood samples were collected after overnight fasting for at least 10 h during the subjects’ medical examination. Plasma samples were stored at −80 °C until analysis. Plasma NO_x_ concentration was determined by the ozone-based chemiluminescence assay [26]. To ensure accurate analysis, during sample collection, light exposure was minimized and distilled water with no NO_2_^−^ content was used. The intra- and inter-assay variability over two months expressed using coefficient of variations was reliably <10%. 

#### 2.2.4. Covariates

Age, body mass index (BMI), cigarette smoking, alcohol consumption, exercise, and total vegetable intake were included in the analyses as covariates. They could be associated with plasma NO_x_ levels [2,27,28,29,30]. Age was calculated from the date of answering the questionnaires and the date of birth. BMI was calculated as body weight (kg) divided by the square of body height (m^2^), which were measured at the medical examination. The definition of smokers that was described in a previous study showing the association between plasma NO_x_ and cigarette smoking was used [29]. The variable of cigarette smoking was “heavy smokers, current smokers with a smoking history of >20 pack years = 2; moderate smokers, current smokers with a smoking history of 1 to 20 pack years = 1; nonsmokers or ex-smokers, others than moderate or heavy smokers = 0”. The variable of alcohol consumption was as follows: Nondrinkers = 0; those who drink once per week or less, but not nondrinkers = 1; those who drink more than once per week = 2. The variable of exercise was “less than once per week = 0; once per week or more = 1”. To control for the effect of NO_x_ from dietary sources, total vegetable intake (g/day) measured by a validated food frequency questionnaire [31] was included in the analyses. In the general population, approximately 70% of the daily dietary exposure to nitrate comes from vegetables [30]. Though venous blood samples were collected after overnight fasting for at least 10 h, NO_x_ from dietary sources might not be fully excreted [5]. The adjustment can also control for the effects of vitamins, minerals, and polyphenols in vegetables on fatigue. 

### 2.3. Statistical Analysis

Differences in continuous variables were compared between men and women using unpaired *t*-tests. Ordinal categorical variables were compared using chi-square test. Then, Pearson’s correlation coefficients were calculated for the correlations between continuous variables, and Spearman’s correlation coefficients were calculated for the correlations that included ordinal categorical variables. Multivariate associations of fatigue with plasma NO_x_ and job strain were assessed with regression analyses. 

Hierarchical regression analyses were employed to examine the hypothesis that NO functioned as an effect modifier in the relationships between job strain and fatigue. In model 1, the variables of job strain, and plasma NO_x_ levels and the product of the variables of job strain and plasma NO_x_ levels were entered as independent variables in a multiple regression model with fatigue as the dependent variable. In model 2, all the aforementioned covariates and the product of the variables of job strain and total vegetable intake were added to the independent variables to control for the possible preventive effect of vegetable intake (*i.e.*, the effect of NO_x_, vitamins, minerals, and polyphenols in vegetables) on fatigue. In accordance with Jaccard *et al.* [32], the continuous variables used as the independent variables were mean-centered. To further examine this interaction, graphic displays of the regression models were also created based on the recommendations described by Cohen *et al.* [33]. The regression lines and predicted values illustrating the significant interactions were constructed from the intercepts, the unstandardized regression coefficients, the mean values, and the standard deviations (SDs). Scores were plotted at the mean, low (1 SD below the mean), and high (1 SD above the mean) values. The scores have been calculated to an accuracy of six figures of decimal points. 

All the *p* values were two-tailed, and *p* < 0.05 was the threshold for significance. All statistical analyses were performed with SPSS version 20.0.

## 3. Results

Participant characteristics according to gender are shown in Table 1. Age, BMI, cigarette smoking, alcohol consumption, exercise, total vegetable intake, job control, job strain, plasma NO_x_, and fatigue were significantly different depending on gender. 

**Table 1:** Participant characteristics according to gender.

	Men (n = 272)			Women (n = 298)			*p* ^a^
	Mean	SD	Range	Mean	SD	Range	
Age (years)	43.6	10.1	20.0–67.7	40.4	10.6	18.6–65.4	<0.001
BMI (kg/m^2^)	23.7	3.5	16.1–37.2	21.6	3.6	14.5–39.7	<0.001
Total vegetable intake (g/day)	125.1	80.3	0–645	144.2	93.0	4–557	0.009
Plasma NO_x_ (μmol/L)	29.4	16.1	7.4–97.3	25.0	13.5	8.5–92.3	<0.001
*Job Content Questionnaire*							
Job demands	32.0	5.5	12–48	32.4	5.6	12–48	0.45
Job control	65.7	10.4	24–90	62.2	10.7	24–90	<0.001
Job strain index ^b^	−0.312	0.087	−0.624–−0.020	−0.283	0.106	−0.684–0.151	<0.001
Fatigue ^c^	7.8	7.2	0–28	11.2	7.6	0–28	<0.001
	n	%		n	%		
*Cigarette smoking*							<0.001
Moderate smoker ^d^	83	30.5		41	13.8		
Heavy smoker ^e^	81	29.8		6	2.0		
*Alcohol consumption*							<0.001
Once per week or less, but not none	64	23.5		128	43.0		
More than once per week	140	51.5		50	16.8		
Exercising once per week or more	107	39.3		61	20.5		<0.001

^a^ Continuous variables were compared using the unpaired *t*-test, and categorical variables were compared using chi-square test; ^b^ Calculated as job demands divided by job control and logarithmically transformed; ^c^ Evaluated using the Profile of Mood States; ^d^ Current smokers with a smoking history of 1 to 20 pack years; ^e^ Current smokers with a smoking history of >20 pack years.

**Table 2:** Correlations ^a^ of the variables used in the study in men.

	1	2	3	4	5	6	7	8	9	10	11
1. Age	1										
2. BMI	0.06	1									
3. Cigarette smoking	0.23 **	−0.04	1								
4. Alcohol consumption	0.10	−0.09	0.13 *	1							
5. Exercise	0.08	−0.06	−0.13 *	0.00	1						
6. Total vegetable intake	0.15 *	0.07	0.05	0.07	−0.00	1					
7. Plasma NO_x_	0.03	0.18*	−0.15 *	−0.04	0.17 *	0.06	1				
8. Job demands	−0.25 **	−0.11	−0.13 *	−0.10	−0.02	0.06	−0.03	1			
9. Job control	0.07	−0.07	0.03	0.20 **	0.09	−0.00	0.07	0.32 **	1		
10. Job strain index	−0.28 **	−0.03	−0.12	−0.22 **	−0.08	0.06	−0.09	0.58 **	−0.57 **	1	
11. Fatigue	−0.20 **	−0.03	−0.09	−0.10	−0.11	−0.02	−0.04	0.45 **	−0.01	0.39 **	1

^a^ Spearman’s correlation coefficients were calculated for the correlations that included variables of cigarette smoking, alcohol consumption, and exercise. For the other correlations, Pearson’s correlation coefficients were calculated; * *p* < 0.05; ** *p* < 0.001.

**Table 3:** Correlations ^a^ of the variables used in the study in women.

	1	2	3	4	5	6	7	8	9	10	11
1. Age	1										
2. BMI	0.20 **	1									
3. Cigarette smoking	−0.05	−0.10	1								
4. Alcohol consumption	0.00	−0.03	0.11	1							
5. Exercise	0.11	0.05	−0.06	0.05	1						
6. Total vegetable intake	0.14 *	0.04	−0.08	−0.08	0.15 *	1					
7. Plasma NO_x_	0.17 *	−0.01	−0.18 *	−0.03	0.25 **	0.21 **	1				
8. Job demands	−0.01	0.07	0.03	0.08	−0.07	0.02	−0.04	1			
9. Job control	0.11	0.13*	−0.04	0.02	0.11	0.06	−0.04	0.14 *	1		
10. Job strain index	−0.11	−0.04	0.07	0.05	−0.13 *	−0.02	0.02	0.64 **	−0.66 **	1	
11. Fatigue	−0.05	−0.03	0.03	0.01	−0.20 **	−0.20 **	−0.16 *	0.42 **	−0.07	0.34 **	1

^a^ Spearman’s correlation coefficients were calculated for the correlations that included variables of cigarette smoking, alcohol consumption, and exercise. For the other correlations, Pearson’s correlation coefficients were calculated; * *p* < 0.05; ** *p* < 0.001.

Correlations of the variables used in the study in men are shown in Table 2. Plasma NO_x_ levels positively correlated with BMI and exercise, and negatively correlated with cigarette smoking with statistical significance. Plasma NO_x_ levels did not significantly correlate with fatigue in men. Correlations of the variables in women are shown in Table 3. Plasma NO_x_ levels positively correlated with age, exercise and total vegetable intake, and negatively correlated with cigarette smoking and fatigue with statistical significance. Job strain and fatigue showed a significant positive correlation in both genders.

Results of multiple regression analyses are shown in Table 4. Plasma NO_x_ levels were significantly associated with fatigue after adjustment for job strain and covariates in women, but not in men. Job strain was significantly associated with fatigue in women (standardized regression coefficient, β = 0.33, 95% confidence interval (CI) 0.23 to 0.44) and in men (β = 0.35, 95% CI 0.24 to 0.47). Both βs were very similar.

**Table 4:** Multiple regression analyses with fatigue ^a^ as a dependent variable.

	Men		Women	
	β ^b^	p	β ^b^	p
Age (years)	−0.06	0.36	0.03	0.59
BMI (kg/m^2^)	−0.03	0.64	−0.03	0.62
*Cigarette smoking*				
Nonsmoker or ex-smoker ^c^ (reference)				
Moderate smoker ^d^	0.06	0.37	−0.05	0.36
Heavy smoker ^e^	−0.07	0.27	0.03	0.57
*Alcohol consumption*				
None (reference)				
Once per week or less, but not none	−0.10	0.16	−0.08	0.21
More than once per week	−0.08	0.24	0.01	0.85
*Exercise*				
Less than once per week (reference)				
Once per week or more	−0.06	0.27	−0.10	0.08
Total vegetable intake (g/day)	−0.05	0.44	−0.16	0.006
Job strain index ^f^	0.35	< 0.001	0.33	< 0.001
Plasma NO_x_ (μmol/L)	0.02	0.77	−0.14	0.02
Adjusted R^2^	0.149 ( *p* < 0.001)		0.161 ( *p* < 0.001)	

^a^ Evaluated using the Profile of Mood States; ^b^ Standardized regression coefficient; ^c^ Others than moderate or heavy smokers; ^d^ Current smokers with a smoking history of 1 to 20 pack years; ^e^ Current smokers with a smoking history of >20 pack years; ^f^ Calculated as job demands divided by job control evaluated using the Job Content Questionnaire and logarithmically transformed.

**Figure 1:**
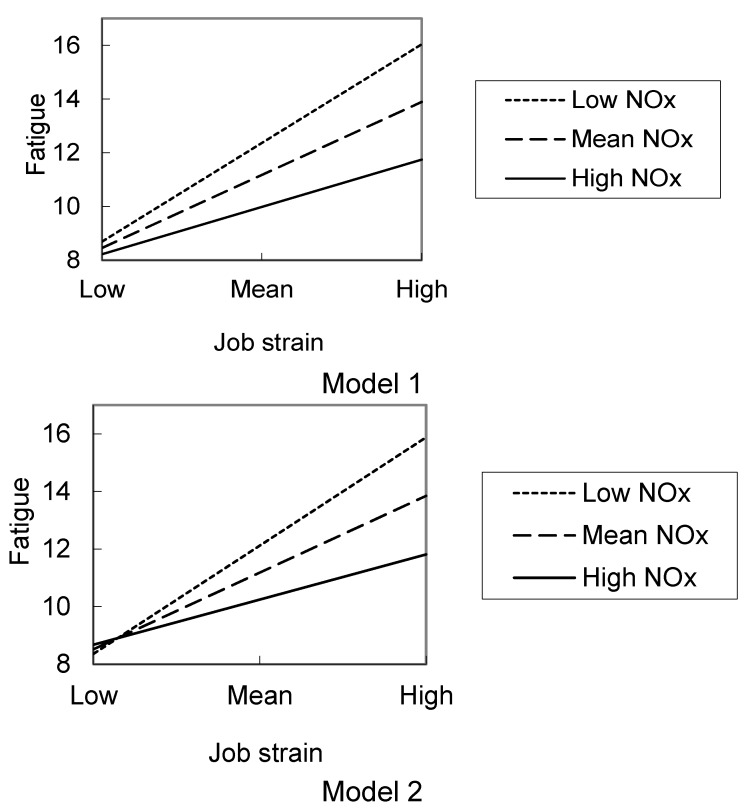
Regression lines and predicted values illustrating the significant interactive effects of job strain and the plasma NO_x_ level on fatigue according to the regression Models 1 and 2 in women.

Results of multiple regression analyses including interactions are shown in Table 5. The interaction between plasma NO_x_ and fatigue significantly contributed to both regression Models 1 and 2 in women, but not in men. The regression lines and predicted values illustrating the significant interactions in both Models 1 and 2 show that, in women, as the level of the job strain worsened, fatigue was exacerbated, but the plasma NO_x_ seemed to buffer the association (Figure 1). 

**Table 5:** Multiple regression analyses with fatigue ^a^ as a dependent variable including interactions.

	Men				Women			
	Model 1		Model 2		Model 1		Model 2	
	β ^b^	p	β^b^	p	β ^b^	p	β ^b^	p
Plasma NO_x_ (μmol/L)	−0.03	0.58	0.00	0.99	−0.16	0.004	−0.12	0.03
Job strain index ^c^	0.41	<0.001	0.37	<0.001	0.36	<0.001	0.35	<0.001
Job strain index ^c^ × plasma NO_x_ (interaction)	−0.07	0.29	−0.05	0.48	−0.12	0.02	−0.14	0.01
Age (years)			−0.06	0.36			0.04	0.53
BMI (kg/m^2^)			−0.03	0.66			−0.03	0.53
Cigarette smoking								
Nonsmoker or ex-smoker ^d^ (reference)								
Moderate smoker ^e^			0.06	0.36			−0.05	0.34
Heavy smoker ^f^			−0.07	0.30			0.03	0.62
Alcohol consumption								
None (reference)								
Once per week or less, but not none			−0.09	0.22			−0.07	0.22
More than once per week			−0.08	0.28			0.01	0.91
Exercise								
Less than once per week (reference)								
Once per week or more			−0.06	0.27			−0.11	0.06
Total vegetable intake (g/day)			−0.05	0.38			−0.17	0.004
Job strain index ^c^ × total vegetable intake (interaction)			−0.06	0.33			0.02	0.73
Adjusted R^2^	0.143 (*p* < 0.001)		0.148 (*p* < 0.001)		0.149 (*p* < 0.001)		0.175 (*p* < 0.001)	

^a^ Evaluated using the Profile of Mood States; ^b^ Standardized regression coefficient; ^c^ Calculated as job demands divided by job control evaluated using the Job Content Questionnaire and logarithmically transformed; ^d^ Others than moderate or heavy smokers. ^e^ Current smokers with a smoking history of 1 to 20 pack years; ^f^ Current smokers with a smoking history of >20 pack years.

## 4. Discussion

NO (evaluated by plasma NO_x_) seemed to be negatively associated with fatigue in women, but not in men. In agreement with this result, in a previous study in which about 70% of the subjects were women, the serum NO_x_ concentration was negatively associated with general fatigue (β = −0.115) after adjustment for age, gender, BMI, and life-time history of major depression [3]. 

Furthermore, NO seemed to buffer the association between job strain and fatigue in women, even after adjustment for potential confounders and the interaction between job strain and vegetable intake. In this study, the relationship between job strain and fatigue in men and women was similar. Thus, the cause for the gender difference seems to involve NO. Previous studies have suggested that there are gender differences in NO production/release or in responses to NO. For example, the rise in plasma NO_x_ concentration in males after KNO_3_ ingestion appeared significantly lower compared with females [19]. Estradiol supplementation improves endothelium-dependent vasodilation in women, probably because of augmented NO production/release, but not in men [20]. Whole-body production of NO was greater in healthy premenopausal women than in men under ambulatory conditions [21]. Future studies on gender differences in NO production/release or in responses to NO may explain the mechanism of the findings in this study. 

This study has some limitations. First, because this study used a cross-sectional design, it was difficult to determine the causal nature of the observed relationships. Longitudinal research is necessary to clarify the causality. Second, because this study used convenience sampling, the results may not be applicable to the entire workforce. However, because subjects were recruited from four entirely different industries and the response rate was more than 50%, some generalizability can be expected. For example, the relationship between job strain and fatigue seen in this study was consistent with previous studies [16,17,18]. This finding of a positive association between age and NO_x_ levels in women, but not in men, is also consistent with a previous study [2]. The negative association between cigarette smoking and plasma NO_x_ shown in this study has also been previously reported [28]. Third, in this study, percentages of smokers, drinkers, and those who exercise were much larger in men than in women. The adjustments for cigarette smoking, alcohol consumption, and exercise using two or three categories might not be sufficient and lead to the failure to detect associations in men.

## 5. Conclusions

In women, NO seemed to be inversely associated with fatigue and to buffer the association between job strain and fatigue, but not in men. 
